# P-1459. Utility of the Pentavalent MenABCWY Meningococcal Vaccine (PenbrayaTM) Within Alternative US Meningococcal Vaccination Schedules

**DOI:** 10.1093/ofid/ofaf695.1645

**Published:** 2026-01-11

**Authors:** Jessica Presa, Steven Shen, Jamie Findlow, Vincenza Snow, Paul Palmer

**Affiliations:** Pfizer, Inc., Collegeville, PA; Pfizer Canada ULC, Kirkland, Quebec, Canada; Pfizer Ltd, Tadworth, England, United Kingdom; Pfizer Vaccines, Collegeville, PA; Pfizer Vaccine Medical Development, Scientific & Clinical Affairs , Collegeville PA, Collegeville, PA

## Abstract

**Background:**

The US meningococcal vaccination schedule – routine vaccination against serogroups A/C/W/Y (MenACWY) at ages 11‒12 and 16 years (y), a 2-dose MenB series based on shared clinical decision making (SCDM) at ages 16‒23 y, and endorsement of Pfizer’s MenABCWY vaccine (Penbraya^TM^) when both vaccines are recommended, is being reevaluated. We assess how MenABCWY clinical data support alternative schedules being considered.

**Figure:**
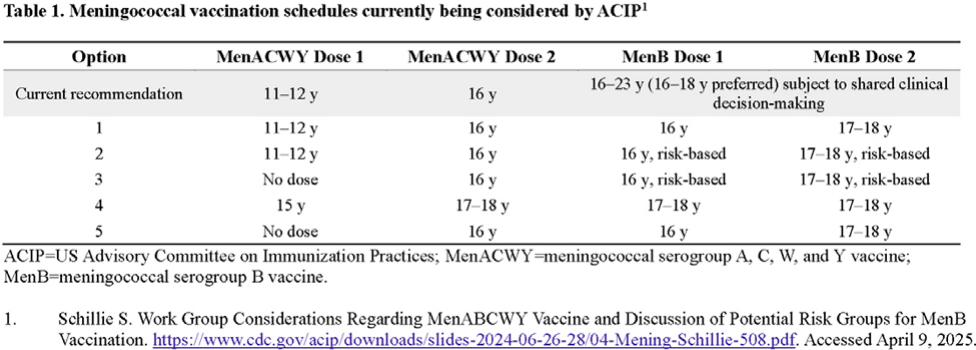

**Methods:**

Alternative meningococcal vaccination schedules were based on materials from recent US Advisory Committee on Immunization Practices meetings. Pfizer’s MenABCWY clinical data were derived from 3 studies that collectively included > 4000 participants aged 10–25 y.

**Results:**

New meningococcal vaccination schedules being considered as of June 2024 (Table 1) include routine or risk-based MenB vaccination due to implementation challenges and poor uptake associated with the existing SCDM recommendation. Pfizer’s MenABCWY vaccine was well tolerated for all schedules (0-, 6-mo; 0-, 12-mo; and 0-, 36-mo); there were no safety concerns. Immune responses were statistically noninferior for 1 MenABCWY dose compared with 1 MenACWY-CRM (Menveo®) dose regardless of previous MenACWY experience and for 2 MenABCWY doses using a 0-, 6-mo schedule compared with 2 MenB-fHbp (Trumenba®) doses. As currently recommended, a MenABCWY dose could replace MenACWY Dose 2 and MenB Dose 1 of proposed schedules both with (options 1, 2, 4) and without (options 3, 5) an earlier MenACWY dose. Compared to the 0-, 6-mo schedule, immune responses for the extended schedules with MenACWY remained robust, and for MenB were higher 1 and 24 months after the last MenABCWY dose suggesting a longer persistence of immune response. Findings support MenABCWY replacement of MenB Dose 2 for all schedule options, expanding serogroup coverage. Option 4 could be reduced to 2 MenABCWY doses at 15 and 17‒18 y, and assuming immune response remains robust for dosing intervals > 3 y, options 1 and 2 could be similarly adjusted.

**Conclusion:**

MenABCWY use in place of separate MenACWY and MenB vaccinations within proposed schedules is supported by clinical data and will reduce the number of injections required, especially for 2-dose, extended-interval MenABCWY schedules spanning the period of elevated risk. Funded by Pfizer.

**Disclosures:**

Jessica Presa, MD, Pfizer Inc: Industry|Pfizer Inc: Stocks/Bonds (Public Company) Steven Shen, MD, PhD, Pfizer Canada ULC: Industry Jamie Findlow, PhD, Pfizer Ltd: Industry|Pfizer Ltd: Stocks/Bonds (Public Company) Vincenza Snow, MD, Pfizer Inc: Industry Paul Palmer, PhD, Pfizer Inc: Employee|Pfizer Inc: Stocks/Bonds (Public Company)

